# 14-3-3 Proteins Buffer Intracellular Calcium Sensing Receptors to Constrain Signaling

**DOI:** 10.1371/journal.pone.0136702

**Published:** 2015-08-28

**Authors:** Michael P. Grant, Alice Cavanaugh, Gerda E. Breitwieser

**Affiliations:** Weis Center for Research, Geisinger Clinic, Danville, Pennsylvania, United States of America; University of Bari Aldo Moro, ITALY

## Abstract

Calcium sensing receptors (CaSR) interact with 14-3-3 binding proteins at a carboxyl terminal arginine-rich motif. Mutations identified in patients with familial hypocalciuric hypercalcemia, autosomal dominant hypocalcemia, pancreatitis or idiopathic epilepsy support the functional importance of this motif. We combined total internal reflection fluorescence microscopy and biochemical approaches to determine the mechanism of 14-3-3 protein regulation of CaSR signaling. Loss of 14-3-3 binding caused increased basal CaSR signaling and plasma membrane levels, and a significantly larger signaling-evoked increase in plasma membrane receptors. Block of core glycosylation with tunicamycin demonstrated that changes in plasma membrane CaSR levels were due to differences in exocytic rate. Western blotting to quantify time-dependent changes in maturation of expressed wt CaSR and a 14-3-3 protein binding-defective mutant demonstrated that signaling increases synthesis to maintain constant levels of the immaturely and maturely glycosylated forms. CaSR thus operates by a feed-forward mechanism, whereby signaling not only induces anterograde trafficking of nascent receptors but also increases biosynthesis to maintain steady state levels of net cellular CaSR. Overall, these studies suggest that 14-3-3 binding at the carboxyl terminus provides an important buffering mechanism to increase the intracellular pool of CaSR available for signaling-evoked trafficking, but attenuates trafficking to control the dynamic range of responses to extracellular calcium.

## Introduction

CaSR is a Family C G protein-coupled receptor activated by extracellular Ca^2+^. CaSR is crucial to establishing and maintaining organismal Ca^2+^ homeostasis, regulating parathyroid hormone synthesis and secretion, intestinal Ca^2+^ uptake, renal Ca^2+^ resorption, and aspects of bone Ca^2+^ uptake and release [1,2]. Beyond its role in Ca^2+^ homeostasis, CaSR is expressed in muscle, epithelia, endothelium and neurons, contributing to cell-type specific regulation of Ca^2+^, lipid signaling including liberation of inositol trisphosphate and diacylglycerol, secretion, differentiation and proliferation [3–5].

CaSR is activated in a highly cooperative manner by serum Ca^2+^, and allosterically modulated by amino acids and small peptides [6]. Despite the constant presence of extracellular Ca^2+^ in all organellar and extracellular environments, CaSR exhibits only weak functional desensitization in the chronic presence of saturating concentrations of extracellular Ca^2+^ [7,8]. The recent demonstration that the sensitivity of CaSR to its agonists and modulators is dependent upon the level of plasma membrane receptors [9] suggests that identifying mechanisms which can be targeted to regulate plasma membrane levels of CaSR may provide novel means of regulating CaSR signaling.

Recent studies from our laboratory have suggested that the ability to signal despite chronic exposure to calcium is mediated by agonist-induced increases in anterograde trafficking of receptors, a process termed Agonist-Driven Insertional Signaling (ADIS) [10–13]. Ongoing signaling via such a mechanism requires an intracellular pool of CaSR sufficient to support a constant level of trafficking from pre-plasma membrane compartments, and may require signaling-induced biosynthesis to maintain the pool. Conversely, such a feed-forward mechanism likely requires a brake or buffering mechanism to constrain trafficking and signaling within physiological limits.

ADIS is modulated by CaSR interactions with 14-3-3 binding proteins at the endoplasmic reticulum [10]. 14-3-3 proteins are a family (7 human isotypes) of small (∼14 kDa) proteins that function as non-covalent dimers, and play broad regulatory roles in cell signaling [14–16]. 14-3-3 binding proteins bind to hundreds of target proteins, and interactions can be regulated by phosphorylation [14]. Interaction with 14-3-3 proteins can affect target protein conformation, interaction with other protein partners, and/or alter subcellular localization [14–17]. 14-3-3 proteins bind at the carboxyl terminus of CaSR at a proximal, arginine-rich motif, ^890^RRSNVSRKR^898^ [18,19]. Mutations which destroy this motif and reduce 14-3-3 protein binding have been identified in patients with Ca^2+^ homeostasis defects, acute pancreatitis or idiopathic epilepsy [20–23]. Replacement of the motif with alanine residues (CaSR-5A, ^890^AASNVSAAA^898^) eliminates 14-3-3 protein binding and increases plasma membrane-localized CaSR [18]. The flanking putative phosphorylation site at S899 regulates 14-3-3 protein interactions with CaSR, i.e., 14-3-3 protein binding is increased in the phosphorylation-deficient mutant S899A and eliminated in the phosphomimetic mutant S899D [18].

In this report, we determined the importance of 14-3-3 protein binding in regulation of CaSR signaling. Outputs of two different signaling pathways, intracellular Ca^2+^ and ERK1/2 phosphorylation, were compared for wt CaSR and 14-3-3 protein binding mutants, and demonstrate significantly higher signaling by the CaSR-5A mutant, which does not interact with 14-3-3 binding proteins. Basal levels of surface receptors for the CaSR-5A mutant were elevated in low extracellular Ca^2+^, and the increase in plasma membrane CaSR-5A induced by elevated extracellular Ca^2+^ was significantly higher than that seen for wt CaSR. Our results demonstrate CaSR interactions with 14-3-3 proteins represent an important buffering mechanism to constrain signaling.

## Materials and Methods

### Cell culture and transfection

Human Embryonic Kidney (HEK) 293 cells (American Type Culture Collection, Manassas, VA) were cultured in MEM supplemented with 10% fetal bovine serum and penicillin/streptomycin in 5% CO_2_ (37°C). Cells were transiently transfected with FuGENE HD (Roche Applied Science, Mannheim, Germany) following the manufacturer’s guidelines.

### DNA constructs

Human wild-type (wt) CaSR and engineered 14-3-3 site mutants (CaSR-5A, in which the arginine-rich motif between residues 890–898 is replaced with five alanine residues; the phosphorylation-resistant mutant CaSR-S899A; and the phosphomimetic mutant CaSR-S899D) having either an amino-terminal FLAG epitope (abbreviated as FLAG-wt, FLAG-mutant) or the FLAG epitope followed by a minimal bungarotoxin binding site (B) and Super Ecliptic pHluorin (SEP) (abbreviated as BS-wt or BS-mutant) were previously reported [10].

### MAPK Assay

HEK293 cells were seeded in 6-well plates, transiently transfected with CaSR cDNAs, and cultured for 48 hours. Medium was then replaced with calcium free DMEM supplemented with 0.5 mM CaCl_2_, 0.1% BSA for an additional overnight incubation. Transfected cells were pre-incubated for 30 min at room temperature (RT) in 0.5 mM Ca^2+^ bath solution containing 0.1% BSA, followed by treatment with the indicated osmolality-matched bath solutions (0.5 or 10 mM Ca^2+^) over a time course of 0–30 min at 37°C, 5% CO_2_. Cells were immediately lysed (on ice) in buffer containing 25 mM HEPES, pH 7.5, 5 mM MgCl_2_, 5 mM EDTA, 1% Triton X100, protease inhibitor cocktail tablet (Roche, EDTA-free), and protein phosphatase inhibitor cocktails 1 and 2 (Sigma-Aldrich), followed by western blotting.

### Tunicamycin treatment

FLAG-CaSR or FLAG-CaSR-5A transiently transfected HEK293 cells were cultured for 2 days in 6 well plates, then treated for 2, 4, 8 or 18 hrs with DMSO/0.5 mM Ca^2+^, 5 μg/ml tunicamycin/0.5 mM Ca^2+^ or 5 μg/ml tunicamycin/5 mM Ca^2+^ in DMEM/0.5% BSA (37°C, 5% CO_2_). Cells were washed with PBS-EDTA on ice, lysed with PBS/5 mM EDTA/0.5% Triton X100 or RIPA buffer containing protease inhibitors (Roche protease inhibitor, mini-EDTA-free tablet) and 100 mM iodoacetamide, and immunoprecipitated with anti-FLAG antibody (M2, Sigma-Aldrich) plus protein G agarose (KPL, Inc.) overnight at 4°C, followed by western blotting.

### Western blotting

MAPK assay lysates or FLAG antibody precipitations were mixed with SDS loading buffer containing dithiothreitol, then incubated for 10 min at 95°C or 30 min at RT, respectively. Samples were run on 4–15% gradient Tris-HCl gels (BioRad) and transferred to PVDF membranes. Blots were probed with anti-phospho-ERK1/2 followed by anti-ERK1/2 antibodies (Cell Signaling, 9101 and 9102, rabbit polyclonals) or anti-CaSR antibody (rabbit polyclonal against LRG epitope (identified as antigenic in [24]), independently custom-generated by Genemed Synthesis, Inc. for us in 2002, first cited in [25]), and species-appropriate secondary antibodies (ECL, HRP-conjugated, GE Healthcare). Protein on blots was visualized with SuperSignal West Pico Chemiluminescence Substrate (Pierce, 34080) on a FUJI Film LAS-4000 Imager, and intensity analysis was done with FUJI Film Multi Gauge v3.1 software.

### Enzyme-linked immunoabsorbance assays (ELISA)

Assays were performed as described previously [18]. Briefly, HEK293 cells were transiently transfected with FLAG-wt or FLAG-tagged mutants (5A, S899A, S899D), cultured for 2 days, then split into 96 well plate for assay on day 3 after transfection. Cells were incubated with 0.5 mM Ca^2+^ bath solution, 0.2% BSA for 30 min at RT, then fixed with 4% paraformaldehyde in PBS (non-permeabilized) or ice cold methanol (permeabilized). Fixed cells were then incubated with anti-FLAG monoclonal antibody conjugated with peroxidase (Sigma-Aldrich), and developed with 3,3’,5,5’-tetramethylbenzidine (Sigma-Aldrich) for 30 min. Reactions were halted with 1 M sulfuric acid, and absorbances read at 450 nm. Similarly processed but untransfected HEK293 cells were used as control, and subtracted from all values.

### Total Internal Reflection Fluorescence Microscopy (TIRFM)

Glass coverslips (22 mm square) were coated with human fibronectin (Millipore, Billerica, MA) prior to HEK293 cell seeding and transient transfection. Three days post-transfection, culture medium was replaced with osmolality matched 0.5 mM Ca^2+^ bath solution containing 0.1% BSA and incubated for 30 min at RT. For each experiment, 1–3 cells were imaged. In some experiments, an automated stage allowed repeated imaging of cells in different fields of view. Cells were plated at low density so we could unequivocally assign fluorescence to an individual cell. Cells were imaged if SEP fluorescence could be visualized in 0.5 mM Ca^2+^ in TIRFM imaging mode, and the entire region of cell contact with the coverslip was in the visual frame. SEP fluorescence in the TIRFM mode provides an estimate of net plasma membrane BS-CaSR. To specifically monitor CaSR internalization, cells were incubated with α-bungarotoxin covalently linked to Alexa Fluor 594 (BgTx-A594; Molecular Probes/Life Technologies; 5 μg/ml for 5 min at RT) in 0.5 mM Ca^2+^ bath solution prior to imaging, or at indicated intervals during experiments. Cells in a sealed perfusion chamber (Warner Instruments, New Haven, CT) were imaged by TIRFM using a 60X, 1.45 NA objective on a Nikon TE2000-E microscope, equipped with Perfect Focus, a TIRF-2 illuminator, and 488 and 594 nm laser lines. TIRFM angles were repetitively adjusted for each wavelength during image acquisition, automated with μManager (National Institutes of Health, USA). Images were captured on a Photometrics CoolSNAP HQ^2^ CCD camera (Tucson, AZ) using appropriate emission filters mounted on a motorized shutter wheel (Ludl, Hawthorne, NY).

### Intracellular calcium imaging

HEK293 cells transiently transfected with the indicated CaSR cDNAs were incubated with 2.5 μM Fura Red-AM with an equal volume of Pluronic, and 2.5 mM probenecid in 0.5 mM Ca^2+^ bath solution for 30 min at 37°C, 5% CO_2_. Cells were then washed and incubated for an additional 30 min at RT prior to imaging on a Nikon TE2000-E microscope using a 594 nm laser line in wide-field mode. Cells were continuously perfused with the indicated bath solutions.

### Confocal imaging

HEK293 cells were seeded on glass coverslips and transiently transfected with BS-wt or BS-5A cDNAs. Cells were incubated in DMEM containing 0.5 or 10 mM Ca^2+^ for 1 hr at 37°C, 5% CO_2_. Following incubation, cells were immediately fixed in 4% paraformaldehyde for 10 min, RT, permeabilized with 0.2% Triton X-100 (10 min), blocked with 5% serum and 0.1% BSA then incubated with monoclonal anti-GFP antibody (Abcam), followed by goat anti-mouse secondary antibody conjugated to Alexa-568. DRAQ5 was used to stain the nucleus. Images were collected on a Leica DM IRE2 confocal laser scanning microscope using a 63X objective and 488, 543 and 633 nm laser lines.

### Image/Data analysis

TIRFM images were analyzed with ImageJ software (National Institutes of Health, USA). Averaged mean surface intensities for regions of interest in individual cells were normalized to the first value in the time series. Background intensities were subtracted using the Subtract Background function in ImageJ. Normalized data from individual cells were averaged over independent experiments, analyzed and plotted with GraphPad Prism v5 (La Jolla, CA). For all experiments, significance was determined by one-way ANOVA followed by Dunnett’s multiple comparisons test, with significance at p< 0.05.

## Results

### Chronic exposure to elevated extracellular calcium increases CaSR expression

**Fig 1:**
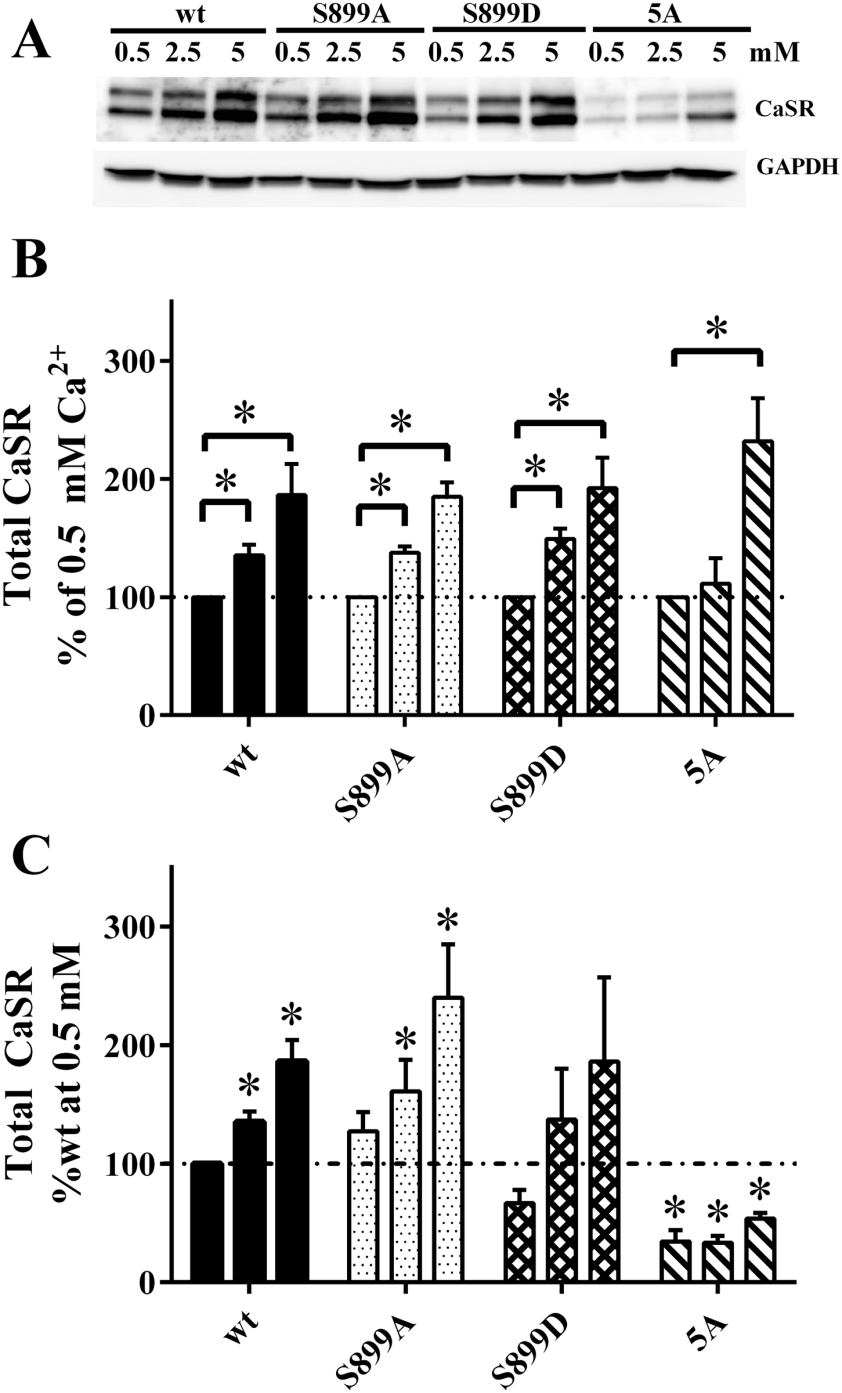
Effect of extracellular Ca^2+^ on net cellular CaSR protein. **A.** HEK293 cells expressing FLAG-wt or 14-3-3 binding mutants as indicated were cultured for 24 hrs (during the period from 48–72 hrs after transfection) in DMEM containing 0.5, 2.5 or 5 mM extracellular Ca^2+^ plus 0.1% BSA. Western blots were probed with anti-CaSR and GAPDH antibodies. **B.–C.** Quantitation of data as in **A**. for n = 4 independent experiments, analyzed by one-way ANOVA followed by Dunnett’s multiple comparisons test, with significance at p < 0.05. Data in **B.** were normalized to expression of each CaSR variant in 0.5 mM Ca^2+^, indicated by dotted line. In **C.** the same data were normalized to total expression of wt CaSR at 0.5 mM Ca^2+^, indicated by dot-dashed line.

ADIS represents a feed-forward mechanism which has been shown to stabilize steady state levels of plasma membrane CaSR and signaling for periods up to 60 min [10]. The long-term consequences of elevated extracellular Ca^2+^ on ADIS are, however, not established. We therefore incubated cells transiently transfected with FLAG-tagged wt CaSR, the 14-3-3 binding site mutant CaSR-5A, and CaSR-S899 mutants for 24 hrs in varying bath [Ca^2+^] over the range from 0.5 to 5 mM to determine the effects on total CaSR expression by western blotting; a representative experiment is in Fig 1A. For wt CaSR and all 14-3-3 site mutants, increasing extracellular Ca^2+^ results in an increase in net cellular CaSR. Cells expressing wt CaSR and the S899 site mutants have substantially higher levels of cellular CaSR at all extracellular [Ca^2+^] when compared to the 5A mutant (Fig 1B and 1C). We quantified levels of net cellular wt CaSR and CaSR-5A in additional experiments. In standard culture medium (~1.4 mM CaCl_2_), there were no significant differences between wt CaSR and CaSR-5A expression levels (wt CaSR, 100%, CaSR-5A, 99.5 ± 9.4%, n = 5). However, prolonged exposure to 0.5 mM Ca^2+^ bath had a significant differential effect on wt CaSR and CaSR-5A protein levels. Net wt CaSR after 18 hrs of 0.5 mM Ca^2+^ was comparable to that at 2 hrs (106.3 ± 20.6%, mean ± S.D. of n = 5 experiments), while CaSR-5A was significantly reduced after 18 hrs in 0.5 mM Ca^2+^ (72.9 ± 22.9%, mean ± S.D. of n = 7 experiments; significantly different from wt CaSR at 18 hrs, by paired t-test, p<0.05). Further, cells expressing the 5A mutant require significantly higher extracellular Ca^2+^ to induce an increase in net CaSR expression (Fig 1B). It should be noted here that results cannot be mediated by transcriptional regulation of mRNA levels, since CaSR and its 14-3-3 binding site variants are being expressed from a plasmid with strong constitutive promoter activity. Further, the coding region of the plasmid does not contain flanking, non-translated regulatory elements found in the native mRNA. Thus, the effects on net CaSR expression reflect post-translational regulation, providing insights into cellular handling of CaSR protein.

**Fig 2:**
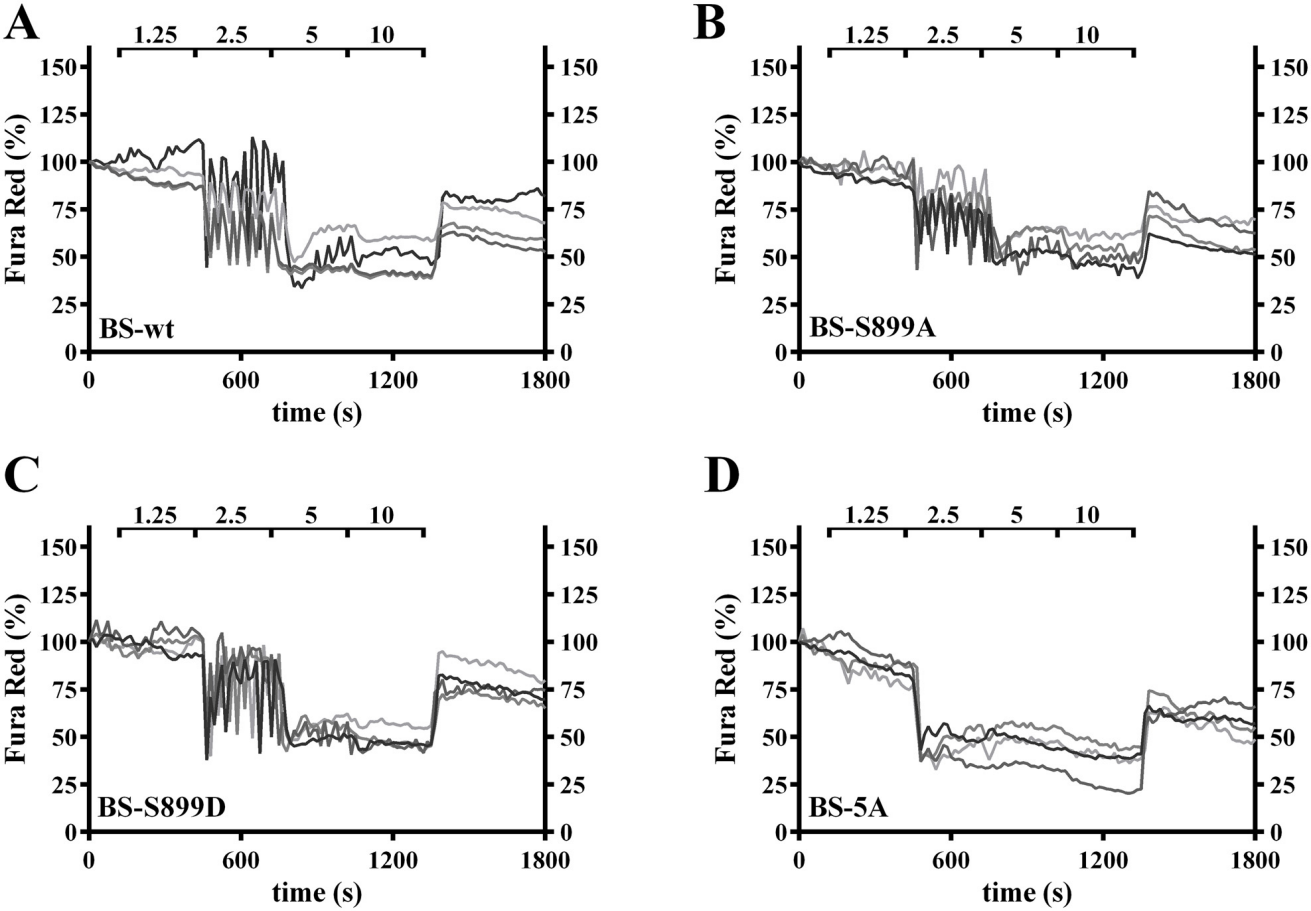
Impact of 14-3-3 protein binding on CaSR-mediated Ca^2+^ signaling responses. **A.–D.** Intracellular Ca^2+^ was assessed by wide-field imaging of Fura Red fluorescence in HEK293 cells expressing BS-wt (**A.**), BS-S899A (**B.**), BS-S899D (**C.**) or BS-5A (**D.**). Each cell is plotted separately with gray scale lines (n = 4 cells). Representative of 3 independent experiments.

We characterized signaling by wt CaSR and the 14-3-3 protein binding site mutants via the two dominant pathways activated by CaSR in most cell types, i.e., Gα_q_-mediated activation of phospholipase C leading to increases in intracellular Ca^2+^, and activation of the MAPK pathway leading to increased phosphorylation of ERK1/2. Changes in cytoplasmic Ca^2+^ were assessed with Fura Red fluorescence, which decreases as intracellular Ca^2+^ increases [10]. Fig 2 illustrates a representative experiment, for wt CaSR and the 14-3-3 protein binding site mutants, in which intracellular Ca^2+^ levels of 4 cells are monitored during sequential increases in extracellular Ca^2+^ from the baseline of 0.5 mM to 1.25, 2.5, 5 and 10 mM. The titration of wild-type CaSR (BS-wt) intracellular Ca^2+^ responses (Fig 2A) confirms previous studies [26,27], with intracellular Ca^2+^ oscillations elicited by 2.5 mM Ca^2+^ and saturation by 5 mM, leading to a steady state increase in intracellular Ca^2+^. Qualitatively similar responses were seen in cells expressing the BS-S899A (Fig 2B) or BS-S899D (Fig 2C) mutants. Intracellular Ca^2+^ responses of BS-5A (Fig 2D), however, are activated at lower extracellular Ca^2+^ concentrations, i.e., 1.25 mM Ca^2+^ elicits some irregular, low amplitude Ca^2+^ oscillations, and a saturating, steady state increase in intracellular Ca^2+^ was achieved by 2.5 mM bath Ca^2+^.

**Fig 3:**
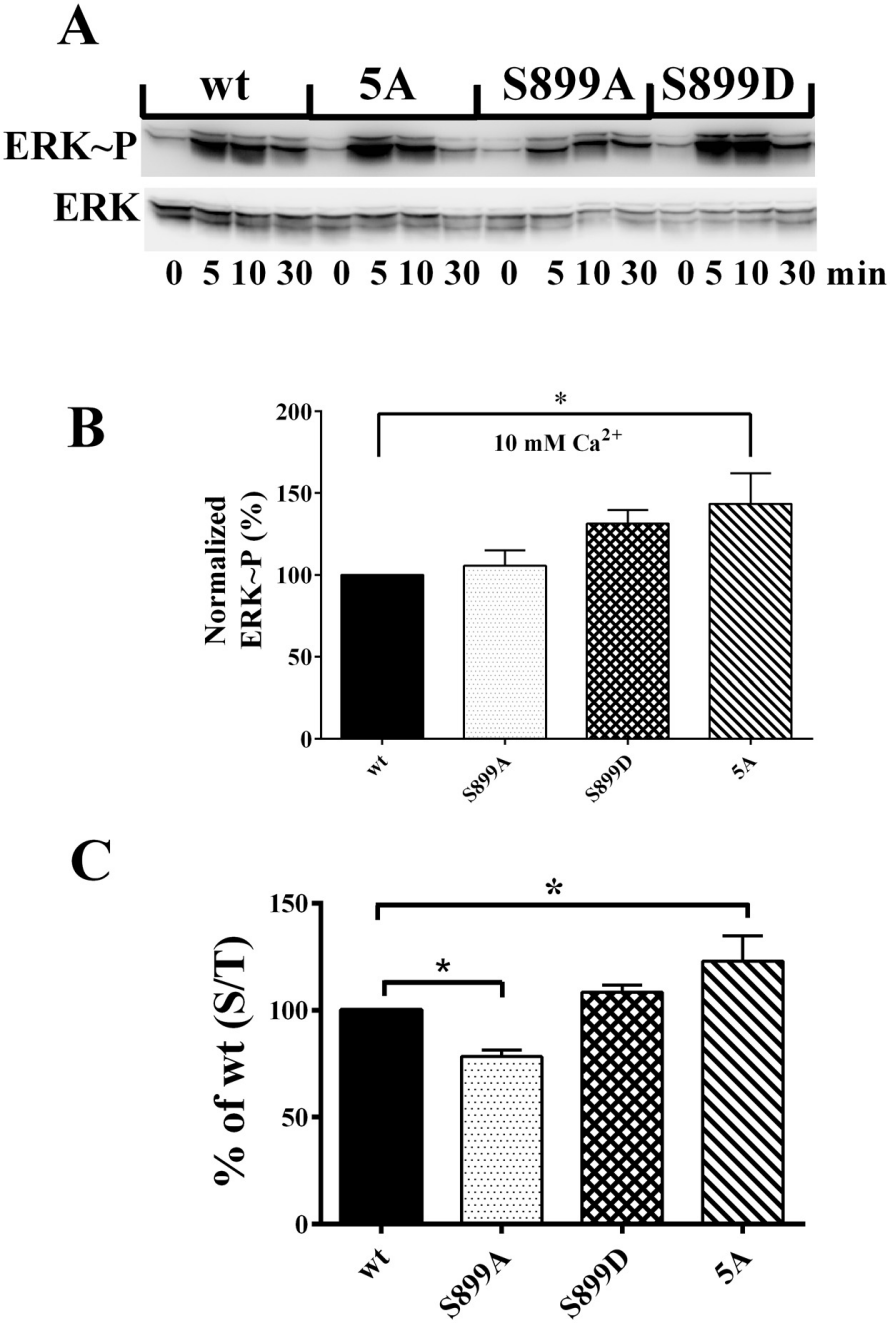
Impact of 14-3-3 protein binding on MAPK signaling of CaSR. **A.** HEK293 cells expressing FLAG-wt, S899A, S899D, or 5A mutants treated with 0.5 (0 time) or 10 mM Ca^2+^ for 5, 10 and 30 min at 37°C. Western blot of lysates were probed with anti-phospho-ERK1/2, then stripped and probed with total ERK antibodies. **B.** Quantitation of ERK1/2 phosphorylation for experiments as in **A.**, for cells treated with 10 mM Ca^2+^ for 10 min. Data were corrected for responses in 0.5 mM, then normalized to responses of wt CaSR (n = 7–10 independent experiments; average ± S.E.M.; analyzed by one-way ANOVA followed by Dunnett’s multiple comparisons test, with significance at *p< 0.05 relative to wt-CaSR). **C.** Net and plasma membrane CaSR were measured by ELISA assay in HEK293 cells expressing BS-wt or 14-3-3 protein binding mutants, as described in Methods. Data are plotted as the percent of surface/total for wt CaSR (% of wt (S/T)) (average ± S.D., n = 4–6 independent experiments; analyzed by one-way ANOVA followed by Dunnett’s multiple comparisons test, with significance at *p< 0.05 relative to wt-CaSR).

In a complementary approach, we used a cell population-based assay to measure CaSR signaling via the MAPK pathway. ERK1/2 phosphorylation in response to 0.5 or 10 mM Ca^2+^ over a 30 min time course was measured. Fig 3A illustrates a blot representative of 5–7 independent experiments. At 0.5 mM Ca^2+^, there were no significant differences in ERK1/2 phosphorylation. However, maximal ERK1/2 phosphorylation was significantly higher for the BS-5A mutant upon stimulation with 10 mM Ca^2+^ for 10 min, when compared to wt or the S899 mutants, as quantified in Fig 3B. For both intracellular Ca^2+^ and ERK1/2 phosphorylation, loss of 14-3-3 binding in the CaSR-5A mutant leads to increased signaling, suggesting that the levels of CaSR-5A at the plasma membrane may be increased, even at 0.5 mM bath Ca^2+^. To test this possibility, we used ELISA assays on cells expressing wt and mutant CaSRs, cultured in 0.5 mM extracellular Ca^2+^. Fig 3C demonstrates that plasma membrane targeting of S899A is significantly reduced relative to wt, while the 5A mutant shows a significant increase in plasma membrane levels relative to wt CaSR. Overall, the results in Figs 1–3 demonstrate that 14-3-3 protein binding contributes to setting plasma membrane levels of CaSR even at low extracellular [Ca^2+^], and loss of 14-3-3 binding in the CaSR-5A mutant permits elevated signaling in response to increases in extracellular Ca^2+^, despite reduced net cellular CaSR levels.

### Sustained ADIS responses of wt CaSR and 14-3-3 binding mutants

**Fig 4:**
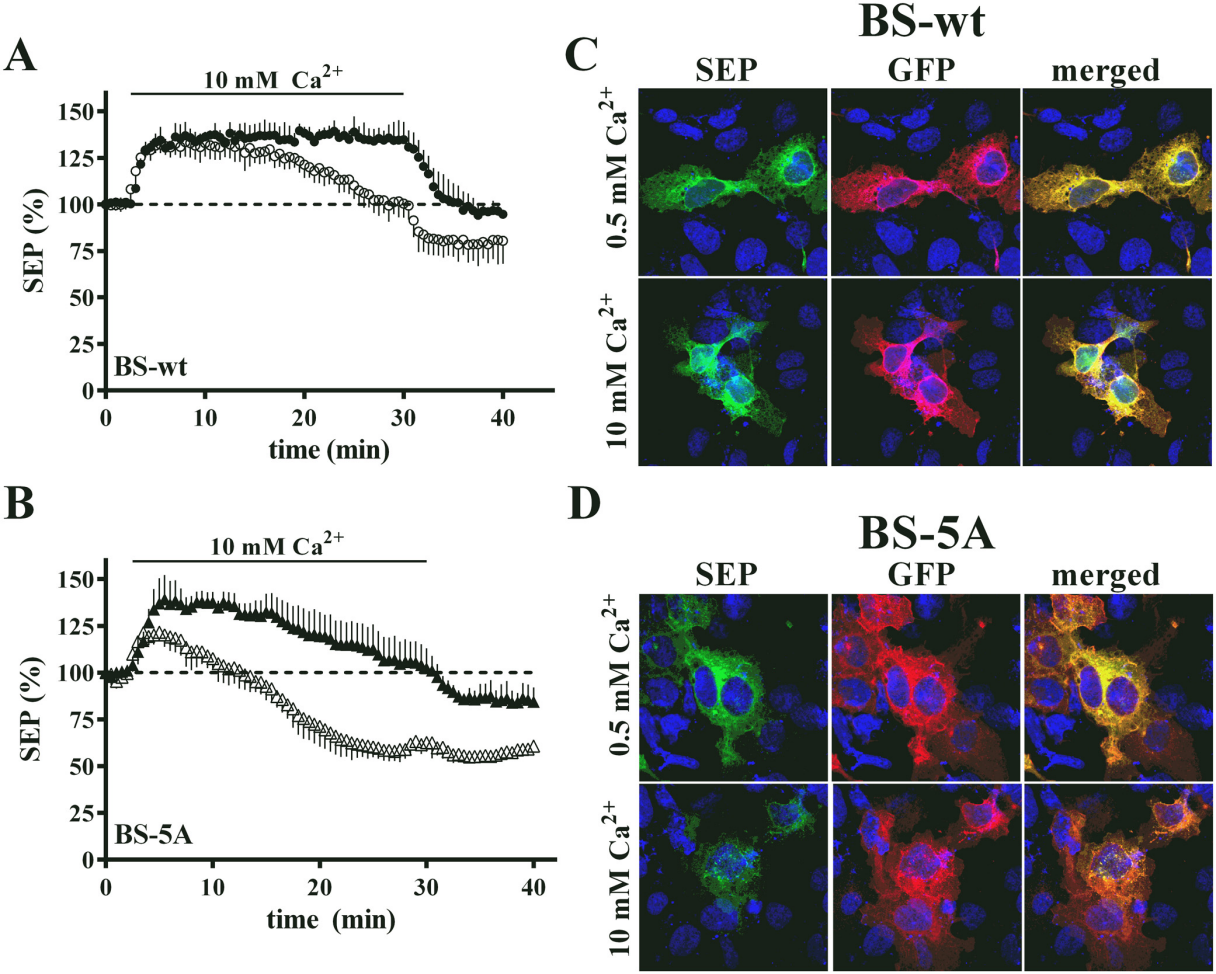
TIRFM responses to prolonged stimulation with 10 mM Ca^2+^. **A.** HEK293 cells expressing BS-wt were stimulated with 10 mM Ca^2+^ in the absence (closed circles) or presence of tunicamycin (open circles) for 30 min, followed by return to 0.5 mM Ca^2+^. Plasma membrane SEP fluorescence was monitored throughout the time course by TIRFM as described in Methods. Data are average ± S.D. of 4 cells. **B.** Experiment as in **A** for cells expressing BS-5A mutant. Data are average ± S.D. of 4 cells. **C.** Cells expressing BS-wt were stimulated with 0.5 or 10 mM Ca^2+^ for 60 min, 37°C, followed by fixation, permeabilization, and staining with anti-GFP antibody (secondary antibody tagged with Alexa-568). Confocal images of SEP and anti-GFP fluorescence are shown along with the merged image. **D.** Experiment as in **C** for cells expressing BS-5A mutant.

Prolonged exposure to elevated extracellular Ca^2+^ requires mobilization of CaSR from intracellular, pre-plasma membrane compartments including the endoplasmic reticulum [10,12]. Because of the differences in net cellular CaSR observed for wt CaSR and the 5A mutant, we compared ADIS of these receptors in response to prolonged exposure to 10 mM extracellular Ca^2+^. For these studies, we compared BS-wt and BS-5A, in cells treated without or with tunicamycin, an inhibitor of cotranslational glycosylation. Previous studies have shown that tunicamycin blocks glycosylation of newly synthesized CaSR, which is a requirement for CaSR exit from the endoplasmic reticulum [10,28]. Cells expressing BS-wt and BS-5A were stimulated with 10 mM Ca^2+^ for 30 min, followed by return to 0.5 mM Ca^2+^. Plasma membrane SEP fluorescence of BS-wt increase rapidly and remained elevated in response to 10 mM Ca^2+^, returning to a level comparable to the pre-stimulation baseline after return to 0.5 mM Ca^2+^, Fig 4A. In contrast, BS-wt cells treated with tunicamycin show the same initial response to 10 mM Ca^2+^, but plasma membrane levels decline over the 30 min exposure, and drop below the initial baseline upon return to 0.5 mM Ca^2+^. Results with BS-5A, Fig 4B, show a similar initial increase and steady state level of plasma membrane SEP fluorescence in response to 10 mM Ca^2+^, but even in the absence of tunicamycin, the response begins to decline after 10 min and is near baseline after 30 min, with a further reduction on return to 0.5 mM Ca^2+^. Treatment of BS-5A cells with tunicamycin reduced the initial response, followed by a rapid decline to 50% below baseline in 10 mM Ca^2+^, with no change after return to 0.5 mM Ca^2+^. For both BS-wt and BS-5A, tunicamycin attenuates the plasma membrane SEP fluorescence responses to prolonged stimulation with 10 mM Ca^2+^, suggesting that maintenance of an elevated steady state level of plasma membrane CaSR requires ongoing recruitment from the endoplasmic reticulum.

TIRFM measurement of net BS-CaSR at the plasma membrane relies on imaging of a GFP variant (SEP) [10]. Prolonged stimulation of cells expressing BS-5A showed a significant reduction in plasma membrane SEP fluorescence (Fig 4B), while MAPK signaling by cells expressing BS-5A remains elevated during a 30 min stimulation with 10 mM Ca^2+^ (Fig 3A and 3B). To reconcile these results, we considered the possibility that the inability of the BS-5A to bind 14-3-3 proteins increases the lability of the intracellular pool, providing insufficient time for maturation of SEP fluorescence, which can require 1–2 hrs [29–32]. To test this possibility, we used a complementary approach. Cells expressing either BS-wt or BS- 5A were cultured for 60 min in 0.5 or 10 mM Ca^2+^ (37°C, 5% CO_2_), then fixed and permeabilized. The SEP moiety was stained with anti-GFP antibody, and confocal imaging was used to assess both intrinsic SEP fluorescence and anti-GFP immunoreactivity. BS-wt had significant SEP fluorescence after 60 min exposure to either 0.5 or 10 mM Ca^2+^, and staining with the anti-GFP antibody was equally robust, showing strong correlation in the merged images (Fig 4C). In 0.5 mM Ca^2+^, BS-5A had strong SEP and anti-GFP fluorescence (Fig 4D), with a merged image comparable to BS-wt. In contrast, there was a significant reduction in BS-5A SEP fluorescence after 60 min in 10 mM Ca^2+^, but anti-GFP staining was robust, leading to reduced colocalization in the merged image (Fig 4D). These results argue that exposure to 10 mM Ca^2+^ rapidly depletes the intracellular pool of BS-5A having mature, i.e., fluorescent, SEP, but net levels of BS-5A are maintained by ongoing biosynthesis, reflected in the anti-GFP staining, consistent with the persistence of BS-5A signaling over the same time period.

**Fig 5:**
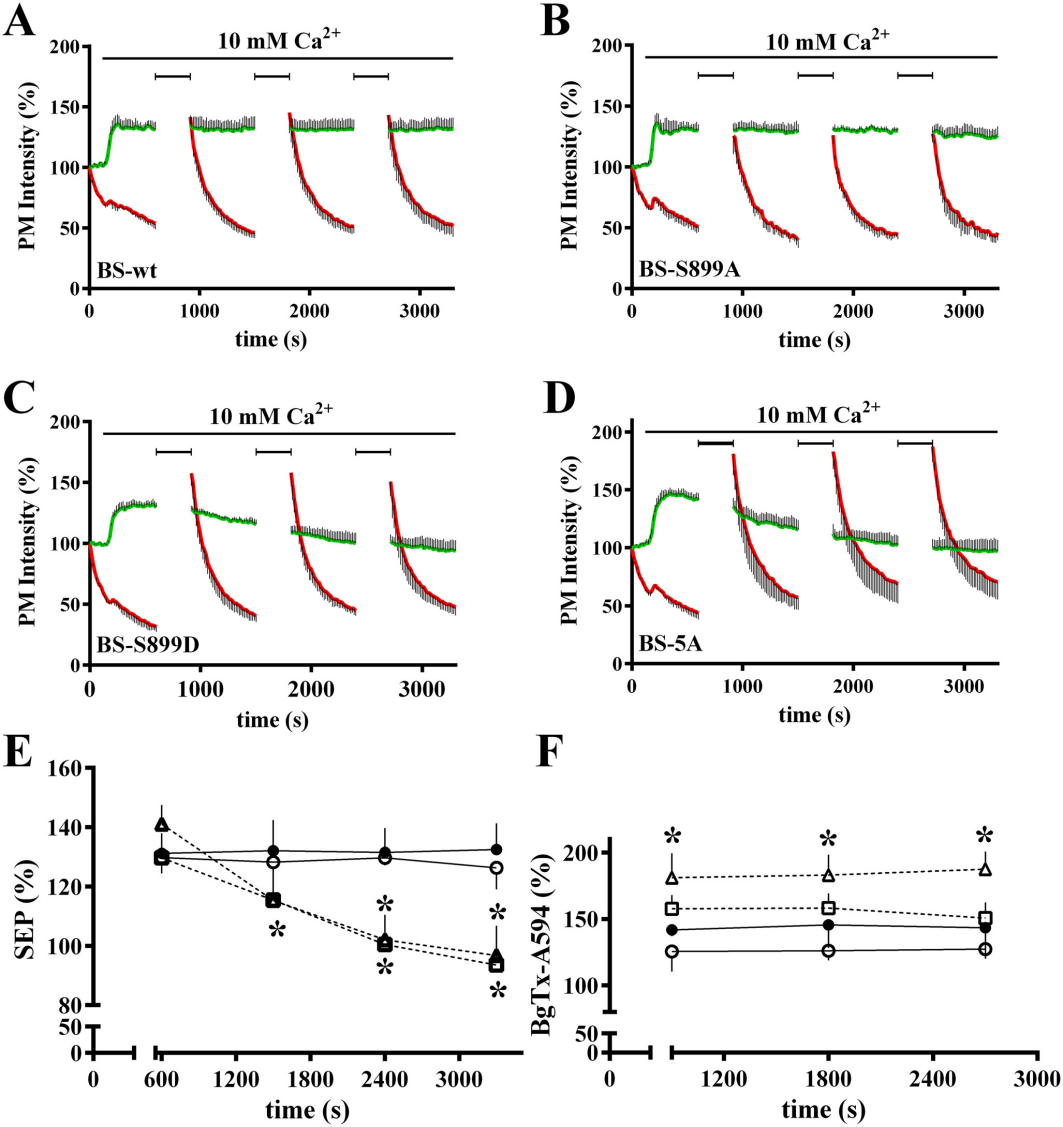
Repeated measures of BgTx-A594 labeling during prolonged stimulation with 10 mM Ca^2+^. Cells expressing BS-wt (**A.**), BS-S899A (**B.**), BS-S899D (**C.**), or BS-5A (**D.**) were labeled with BgTx-A594 prior to imaging, and then imaging was begun in 0.5 mM Ca^2+^. Stimulation with 10 mM Ca^2+^ (indicated by bar) was initiated. Imaging was halted periodically (indicated by brackets) for labeling with BgTx-A594, then resumed. Plotted are the averages ± S.D. of SEP (green) and BgTx-A594 (red) fluorescence for n = 4 cells, representative of 3 independent experiments. **E.** Averaged SEP fluorescence just prior to each BgTx-A594 labeling period for cells illustrated in **A–D** is plotted. BS-wt (closed circles), BS-S899A (open circles) are drawn with solid lines, while mutants which do not bind 14-3-3 proteins are drawn with dotted lines (BS-S899D (open squares), BS-5A (open triangles)). **F.** Peak BgTx-A594 after each labeling period in 10 mM Ca^2+^, with symbols and statistical analysis as in **E.** For **E.** and **F.**, data were analyzed using one-way ANOVA followed by Dunnett’s multiple comparisons test, with significance at *p < 0.05 relative to BS-wt CaSR at the same time point.

The BS-CaSR chimera contains not only SEP, but a bungarotoxin binding site which we have used to rapidly label plasma membrane-localized CaSR to monitor endocytosis [10]. The bungarotoxin binding site is a linear epitope recognized by fluorescent adducts of bungarotoxin. We reasoned that repeated, short labeling periods with fluorescent bungarotoxin would provide a means of monitoring net plasma membrane BS-CaSR independent of SEP fluorescence. Cells expressing BS-wt (Fig 5A), BS-S899A (Fig 5B), BS-S899D (Fig 5C) and BS-5A (Fig 5D), were labeled with BgTx-A594 at the beginning of the experiment in 0.5 mM Ca^2+^, then TIRFM imaging was begun, and cells were exposed to 10 mM Ca^2+^. At three intervals during sustained exposure to elevated extracellular Ca^2+^ (marked by bars), imaging was briefly halted to label cells with BgTx-A594. TIRFM imaging of both SEP and BgTx-A594 fluorescence was then resumed. Data are presented as the average ± S.D. of 4 cells, and representative of 3 independent experiments. Plasma membrane BS-wt SEP fluorescence (green) was constant during the 55 min experiment, and each application of BgTx-A594 elicited a peak followed by mono-exponential decay of fluorescence indicative of endocytosis (Fig 5A). Similar results were obtained with BS-S899A (Fig 5B), i.e., the steady state level of plasma membrane BS-S899A was constant over the recording period, and each round of BgTx-A594 labeling generated a comparable endocytic response. For the two mutants that do not bind 14-3-3 proteins, results were different. Both BS-S899D (Fig 5C) and BS-5A (Fig 5D) had an initial increase in net plasma membrane receptors in 10 mM Ca^2+^, but levels of SEP fluorescence slowly declined during prolonged exposure, reaching baseline after ≈40 min. In contrast, BgTx-A594 labeling was constant throughout the exposure to 10 mM Ca^2+^, and showed a higher level of plasma membrane-localized receptors than SEP fluorescence. Detailed analysis of SEP and BgTx fluorescence time courses show the differences, i.e., BS-wt and BS-S899A, which bind 14-3-3 proteins, show stable SEP fluorescence, while BS-S899D and BS-5A, which are deficient in 14-3-3 protein binding, show a steady decline (Fig 5E). In contrast, BS-wt and all mutants showed constant peak levels of BgTx labeling throughout exposure to 10 mM Ca^2+^, with comparable levels of labelling for BS-wt and the two phosphorylation site mutants, and significantly higher labelling of BS-5A compared to BS-wt (Fig 5F). Responses to each BgTx-A594 labeling period were fitted with single exponential decay curves and compared. Only the extrapolated peak response of BS-5A differed from BS-wt (Fig 5F). Since BgTx-A594 time courses reflect endocytosis of receptors localized to the plasma membrane during the labeling period, results indicate that differences in net plasma membrane levels are not due to altered endocytosis rates but to differences in anterograde trafficking.

**Fig 6:**
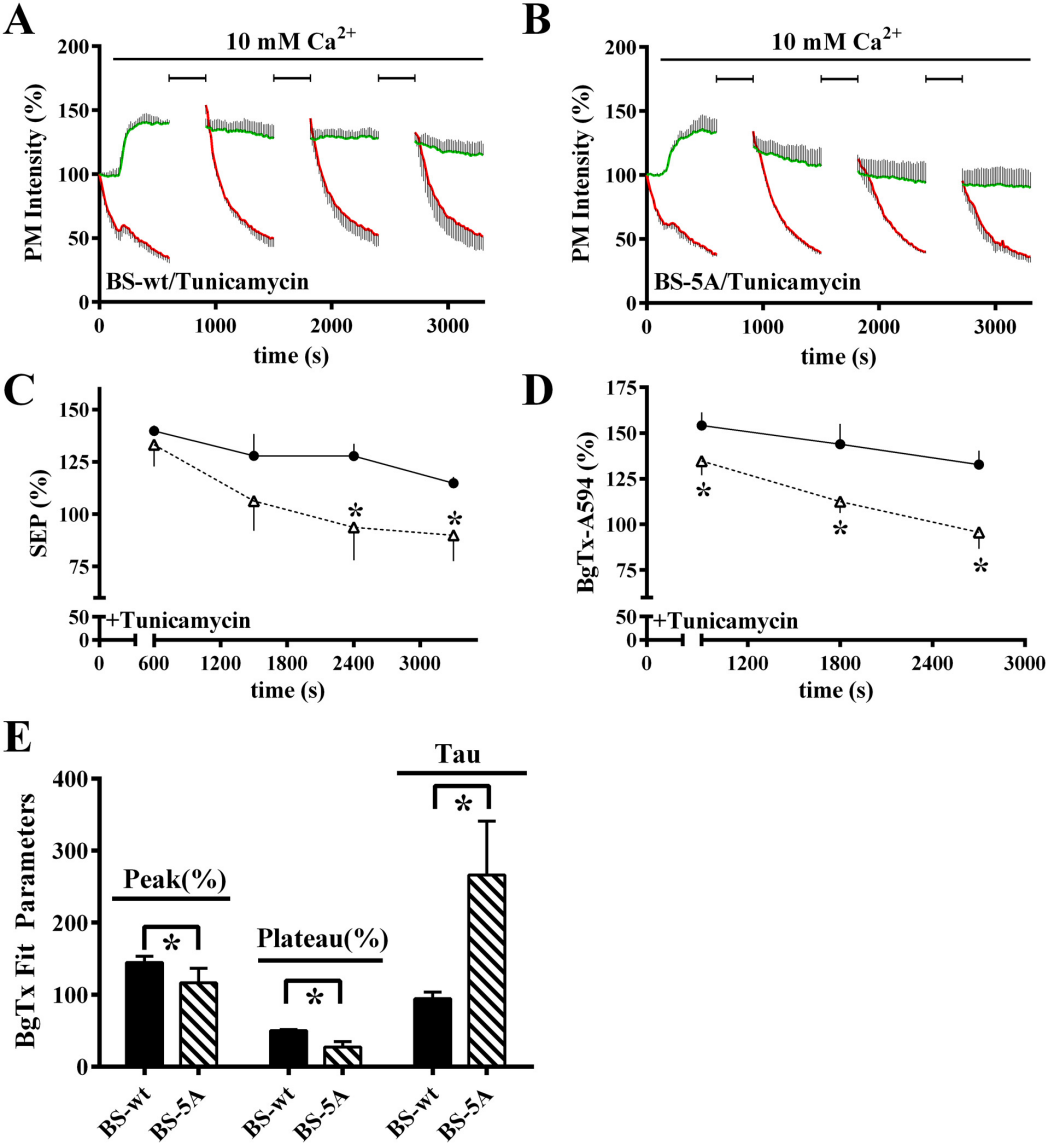
Tunicamycin reduces SEP and BgTx-A594 responses of BS-wt and BS-5A. **A.–B.** Cells expressing BS-wt (**A.**) or BS-5A (**B.**) were labeled with BgTx-A594 in 0.5 mM Ca^2+^, then recording of SEP and BgTx-A594 fluorescence at the plasma membrane was begun using TIRFM. Imaging was interrupted periodically (indicated by brackets) for relabeling with BgTx-A594 during stimulation with 10 mM Ca^2+^ (indicated by bar). Cells were exposed to 5 μg/ml tunicamycin for 60 min prior to imaging and throughout the experiment. Plotted are the averages ± S.D. of SEP (green) and BgTx-A594 (red) fluorescence of n = 4 cells, representative of 3 independent experiments. **C.** Averaged SEP fluorescence just prior to each BgTx-A594 labeling period for cells illustrated in **A.–B.** are plotted. BS-wt (closed circles), and BS-5A (open triangles), for n = 4 cells, plotted as average ± S.D. **D.** Peak BgTx-A594 after each labeling period in 10 mM Ca^2+^. Symbols as described in **C.** For **C.** and **D.**, data were analyzed using one-way ANOVA followed by Dunnett’s multiple comparisons test, with significance at *p < 0.05 relative to BS-wt CaSR at the same time point. **E.** Individual BgTx-A594 responses at 10 mM Ca^2+^ within each experiment of **A–B.** were fitted with single exponential decays, and peak, plateau and decay time constants calculated and normalized to the first response for each experiment. Normalized fit parameters for BgTx-A594 responses in 10 mM Ca^2+^ for BS-wt (black bars) and BS-5A (hatched bars) are plotted. BS-5A responses were significantly different from BS-wt (analyzed by paired t-test, significance at *p< 0.05 relative to BS-wt).

We hypothesized that receptors which bind 14-3-3 proteins are stabilized in the endoplasmic reticulum for a period sufficient to permit SEP chromophore maturation, i.e., formation of the covalent bond which results in SEP fluorescence, while receptors which do not bind 14-3-3 proteins are more likely to exit the endoplasmic reticulum prior to such maturation. To test this hypothesis, we repeated the experiment of Fig 5 in cells treated with tunicamycin prior to and during stimulation with 10 mM Ca^2+^. Tunicamycin caused unstable and slowly declining plasma membrane SEP fluorescence for BS-wt in response to 10 mM Ca^2+^, accompanied by a time-dependent decline in BgTx-A594 labeling (Fig 6A), indicating that newly synthesized BS-wt contributes to the stability of the steady state response under control conditions (compare with Fig 5A). The response of BS-5A was more pronounced (Fig 6B), with a rapid decline in both the net plasma membrane SEP fluorescence and BgTx-A594 labeled fraction, implying that only BS-5A which was synthesized and had matured prior to tuncamycin addition contributed to the ADIS response. Fig 6C and 6D illustrate the plasma membrane SEP and BgTx-A594 peak responses, respectively, over time in 10 mM Ca^2+^ in the presence of tunicamycin. BS-5A responses were significantly different from BS-wt in all assessed parameters (peak (%), plateau (%), and time constant (tau)) (Fig 6E). The requirement for newly synthesized receptors in ongoing ADIS magnifies the differences between the intracellular, signaling-mobilizable pools of BS-wt and BS-5A.

### Net levels of mature CaSR are maintained by coupling anterograde trafficking to biosynthesis

**Fig 7:**
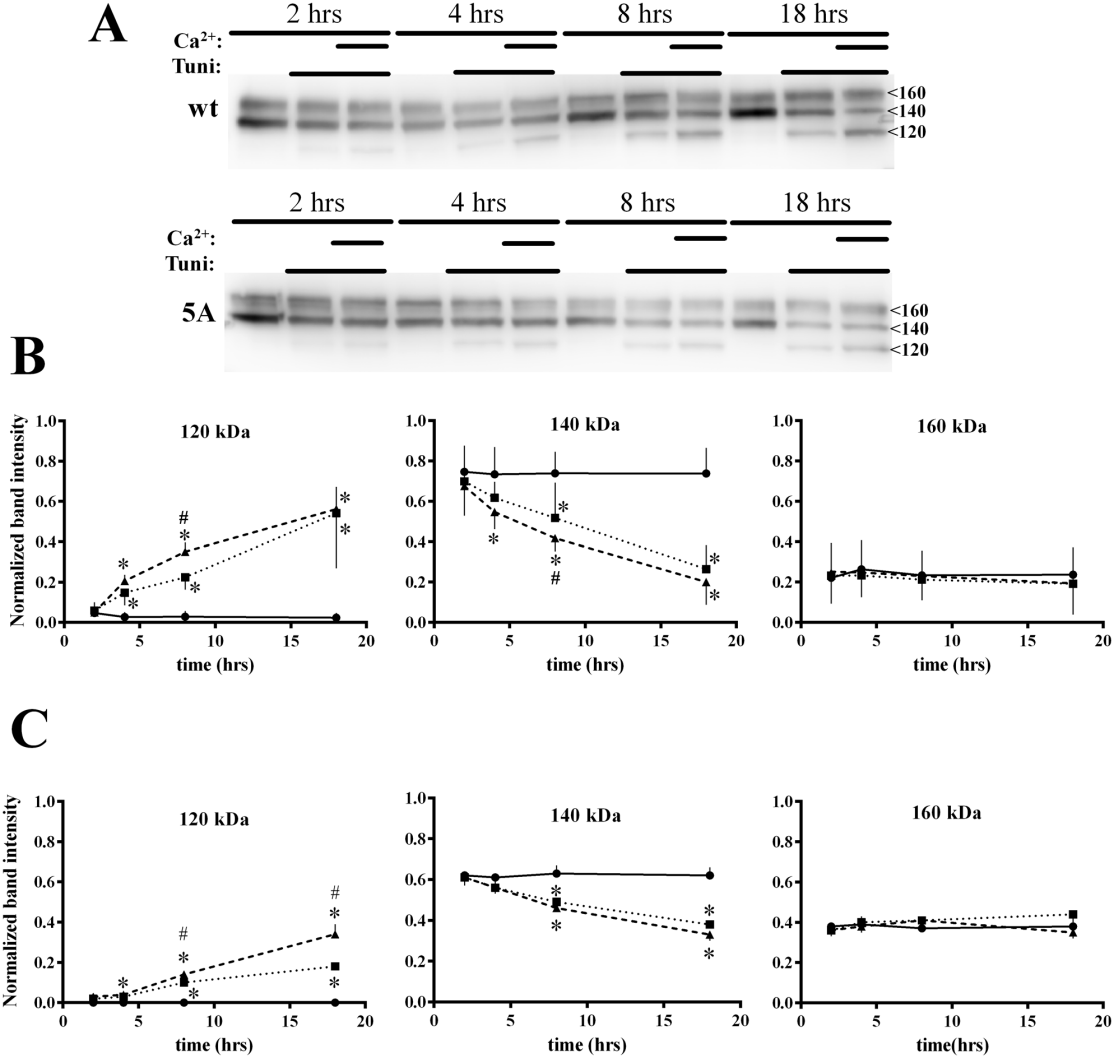
Net cellular CaSR levels show correlates of the ADIS response. **A.** Western blot of HEK293 cells expressing FLAG-CaSR or FLAG-CaSR-5A, treated for indicated times with DMSO (vehicle)/0.5 mM Ca^2+^, 5 μg/ml tunicamycin/0.5 mM Ca^2+^ (long thin bar) or tunicamycin/5 mM Ca^2+^ (short, bracketed thick bars). Cells were lysed at the indicated times and processed for western blotting as indicated in Methods. **B.** Quantitation of the 120, 140 and 160 kDa bands over the experimental time course for 5–7 experiments as in **A.** for wt CaSR. DMSO (black circles, solid line), tunicamycin (black squares, dotted line), and tunicamycin plus Ca^2+^ (black triangles, dashed line) are plotted. **C.** Quantitation of the 120, 140 and 160 kDa bands over the experimental time course for 5–7 experiments as in **A** for the 5A mutant. Control (black circles, solid line), tunicamycin (black squares, dotted line), and tunicamycin plus Ca^2+^ (black triangles, dashed line) are plotted. For **B.** and **C.**, * indicates significant difference from DMSO (p<0.05); # indicates a significant difference between tunicamycin/0.5 mM Ca^2+^ and tunicamycin/5 mM Ca^2+^ (p<0.05).

We next determined whether extracellular Ca^2+^ causes net changes in cellular CaSR, and whether this is altered by mutations at the 14-3-3 protein binding site. Cells expressing FLAG-wt or FLAG-5A were treated with DMSO/0.5 mM Ca^2+^, tunicamycin in 0.5 or 5 mM Ca^2+^ for 2, 4, 8 or 18 hrs, then lysed and subjected to western blotting. Fig 7A illustrates representative blots. The time courses of change in band intensities for each form were quantified for wt Figs 7B and 5A mutant (Fig 7C) from 7–9 independent experiments. The most striking finding is that cells maintain a constant level of the maturely glycosylated 160 kDa form at the expense of the 140 kDa immaturely glycosylated form. Also, the ratio of immature to mature glycosylated forms remains constant under control conditions, but differs between wt CaSR (~80% immature, ~20% mature) and the 5A mutant (~60% immature, ~40% mature) (significance estimated by paired t-tests comparing same timing and condition between wt-CaSR and CaSR-5A, *p<0.05). Results are consistent with the higher level of 5A mutant signaling and plasma membrane receptor density as characterized in Figs 1–3. The present results argue that a greater proportion of total 5A mutant is mature, i.e., resides in Golgi and post-Golgi compartments. Tunicamycin treatment reveals the dynamics of CaSR regulation. The cellular level of the 160 kDa form is maintained at the expense of the 140 kDa form. Ongoing cellular synthesis of CaSR is reflected in the time-dependent increase in the unglycosylated 120 kDa form, which is trapped in the endoplasmic reticulum [10,28]. Thus, in the absence of tunicamycin, the constant level of 140 kDa form is maintained by ongoing synthesis, replacing CaSR which is released to the secretory pathway.

Exposure to 5 mM Ca^2+^ in the presence of tunicamycin further accelerates loss of the 140 kDa form and increases production of the 120 kDa form for wt CaSR. Comparison of the dynamics of the 120 and 140 kDa forms for wt and the 5A mutant demonstrates the importance of 14-3-3 binding in controlling CaSR synthesis and trafficking. Stimulation of wt CaSR with 0.5 mM Ca^2+^ leads to a significantly greater loss of the 140 kDa and synthesis of the 120 kDa forms at 8 hrs (paired t-tests, *p<0.05 relative to DMSO, #p<0.05 relative to 0.5 mM Ca^2+^), although this difference is lost by 18 hrs. The 5A mutant, on the other hand, shows a significant lag in induction of the 120 kDa form, although addition of 5 mM Ca^2+^ doubles the rate of synthesis (significance estimated by paired t-tests, *p<0.05 relative to no DMSO, #p<0.05 relative to 0.5 mM Ca^2+^). The rate of loss of the 140 kDa form of the 5A mutant is not sensitive to extracellular Ca^2+^, Fig 7C. Overall, these differences are reflected in the more rapid decrease in net 5A mutant over the 18 hr time course, eg., Fig 7A, and the inability to build net CaSR levels comparable to wt CaSR, as seen in Fig 1A.

## Discussion

This report characterizes the role of 14-3-3 proteins in regulating CaSR signaling in elevated extracellular Ca^2+^. An inability to bind 14-3-3 proteins leads to higher basal levels of plasma membrane CaSR despite a reduction in net cellular CaSR. Signaling is commensurate with plasma membrane levels of receptors, i.e., we observed left-shifted sensitivity to extracellular Ca^2+^ for activation of steady changes in intracellular Ca^2+^, as well as increased Ca^2+^-stimulated ERK1/2 phosphorylation. Loss of 14-3-3 binding did not, however, result in complete loss of agonist-mediated increases in plasma membrane CaSR. The ADIS response, as monitored by periodic BgTx-A594 labelling, is significantly higher for the CaSR-5A mutant than wt CaSR at elevated extracellular Ca^2+^. Converse results, observed for the CaSR-S899A mutant, argue that tight binding of 14-3-3 proteins leads to enhanced intracellular retention of receptors, and thus reduced signaling. Independent confirmation of these differences is clearly demonstrated by the reduced CaSR-S899A and elevated CaSR-5A localizations at the plasma membrane in 0.5 mM Ca^2^, as measured by ELISA. Cellular Ca^2+^ homeostasis is tightly controlled, and its dysregulation can contribute to pathologies ranging from diabetes to Parkinson’s disease [33–36]. For cells expressing CaSR, tight regulation of plasma membrane levels may be required to maintain intracellular Ca^2+^ within physiological limits. Mutations in the 14-3-3 protein binding region of CaSR, including R896H and R898Q, have been identified in patients with pancreatitis [20] and idiopathic epilepsy syndrome [21], and a large in-frame deletion at the carboxyl terminus which eliminates the 14-3-3 binding site causes autosomal dominant hypocalcemia [22,23]. We have shown that these mutations increase plasma membrane targeting and signaling [18], supporting 14-3-3 protein binding as an important determinant of physiological regulation of CaSR signaling. The present results define the mechanism of 14-3-3 proteins regulation of CaSR signaling, i.e., binding of CaSR to 14-3-3 proteins controls initial steps in CaSR trafficking to the plasma membrane, limiting release from the endoplasmic reticulum to constrain signaling.

One of the most striking results from the present study is the evidence for enhanced lability of the intracellular, mobilizable pool of CaSR caused by loss of 14-3-3 protein binding. We used the relative plasma membrane SEP and BgTx-A594 fluorescence intensities to characterize this lability and its effect on the magnitude of ADIS responses for BS-wt and 14-3-3 protein binding mutants. When normalized plasma membrane SEP and BgTx-A594 fluorescence intensities are comparable, then the dwell time in intracellular, pre-plasma membrane compartments must be sufficient for full maturation of the SEP chromophore. Estimates of chromophore maturation rates for GFP variants range from hours for wild-type GFP to less than an hour for enhanced folding mutants [29–32], arguing that the dwell time for individual BS-CaSR molecules (wt or S899A) in the endoplasmic reticulum post-synthesis can be significant under physiological conditions. In contrast, the BS-CaSR mutants which do not bind 14-3-3 proteins show significantly lower SEP compared to BgTx-A594 fluorescence during ongoing signaling, implying that a significant fraction of plasma membrane receptors were mobilized from the endoplasmic reticulum prior to chromophore maturation. In an earlier study, we used plasma membrane SEP fluorescence at elevated extracellular Ca^2+^ to conclude that both wt CaSR and CaSR-5A are subject to additional regulatory interactions which determine maximal trafficking rates [10]. In this report, however, we clearly demonstrate that the lability of the CaSR-5A intracellular pool makes SEP fluorescence a poor marker for net plasma membrane targeting of some CaSR mutants. Using rapid labeling with BgTx-A594 demonstrates the significantly larger ADIS response elicited by elevated extracellular Ca^2+^. While the original conclusion remains valid, i.e., there are additional as yet undefined determinants of the ADIS response, the present results establish the importance of 14-3-3 proteins in decreasing the lability of the intracellular, ADIS-mobilizable pool of CaSR. The complete lack of ADIS response of the truncation mutant, CaSRΔ868 [10], and the enhanced ADIS responses observed here for CaSR-5A argue that additional determinants controlling CaSR trafficking to the plasma membrane reside in the distal carboxyl terminus of CaSR. Further studies will be required to identify additional interacting proteins, and determine the hierarchy of CaSR trafficking regulation by these protein partners.

A second major result of these studies is the requirement for ongoing biosynthesis to maintain CaSR signaling during prolonged exposure to elevated extracellular Ca^2+^. Tunicamycin, which rapidly blocks endoplasmic reticulum exit of newly synthesized CaSR [10,28], induces concordance between SEP and BgTx-A594 signals for both BS-wt and BS-5A. Ongoing biosynthesis, therefore, maintains the steady state levels of plasma membrane CaSR in elevated extracellular Ca^2+^. Results suggest that CaSR expression levels are modulated by ambient extracellular Ca^2+^ in a feed-forward mechanism, i.e., increased extracellular Ca^2+^ activates CaSR signaling, which increases net cellular CaSR levels, stabilized by 14-3-3 protein binding. An inability to bind 14-3-3 proteins does not, paradoxically, attenuate signaling, but enables rapid targeting of newly synthesized receptors to the plasma membrane, leading to increased coupling between biosynthesis and signaling.

Finally, biochemical approaches demonstrate that prolonged stimulation with elevated extracellular Ca^2+^ also increases net cellular levels of CaSR, ensuring that the intracellular, ADIS-mobilizable pool is sufficient to maintain signaling. It should be noted that the present study interrogates only translational and post-translational mechanism(s) contributing to establishment and maintenance of cellular levels of CaSR, since transcription is driven by the vector CMV promoter. Prolonged stimulation *in vivo* may be additionally regulated at the transcriptional level. We here demonstrate that mechanism(s) contributing to regulation of CaSR protein are poised to maintain a constant level of maturely glycosylated CaSR, at the expense of the immaturely glycosylated, endoplasmic reticulum-localized form. In this context, the net level of the 160 kDa form is not equivalent to plasma membrane CaSR levels, but rather is the sum of CaSR localized to Golgi, plasma membrane and post-plasma membrane sites. Nevertheless, the most striking result of these studies is that cellular biosynthesis and trafficking of CaSR is poised to maintain plasma membrane levels of CaSR despite alterations in cell signaling and/or cell stress, induced in these studies by tunicamycin [37,38]. These results have practical implications, since they argue that siRNA-mediated depletion of cellular CaSR can be potentiated by culture in elevated extracellular Ca^2+^, to facilitate depletion of pre-plasma membrane pools of CaSR. Finally, prolonged exposure to elevated extracellular Ca^2+^ leads to net cellular increases in CaSR, mediated by an increased rate of CaSR biosynthesis [39] counterbalanced by a constant rate of endocytosis and degradation [10,40]. Prolonged exposure to agonist leads to down-regulation of many G protein-coupled receptors [41–48]. In contrast, strong coupling between signaling and biosynthesis plus a substantial intracellular, mobilizable pool may be required for CaSR, which must be able to respond to small changes in extracellular Ca^2+^ superimposed on a constant baseline of Ca^2+^ [1,2].

## Conclusions

In summary, these studies use novel approaches to demonstrate that 14-3-3 protein binding at the endoplasmic reticulum stabilizes and decreases the lability of the intracellular, ADIS-mobilizable pool of CaSR. CaSR binding to 14-3-3 proteins supports ongoing CaSR signaling while maintaining tight control of the dynamic range of signaling responses.
